# Fatalities following DMT use: two case reports and a review of the literature

**DOI:** 10.1093/jat/bkaf064

**Published:** 2025-07-09

**Authors:** Jade Pullen, Robert Moore, Rebecca Wood, Edmund Rab, Lewis Couchman, Caroline S Copeland

**Affiliations:** National Programme on Substance Use Mortality, London, United Kingdom; Toxicology Department, Royal Sussex County Hospital, Brighton, BN2 5BE, United Kingdom; Toxicology Department, Royal Sussex County Hospital, Brighton, BN2 5BE, United Kingdom; Specialised Clinical Chemistry, Sheffield Teaching Hospitals, Sheffield, S10 2JF, United Kingdom; Analytical Services International Ltd, St George’s, University of London, London, SW17 0QT, United Kingdom; National Programme on Substance Use Mortality, London, United Kingdom; Institute of Pharmaceutical Science, King’s College London, London, SE1 9NH, United Kingdom

## Abstract

*N, N*-Dimethyltryptamine (DMT) is a hallucinogen found in the South American *Psychotria viridis* plant and is the major psychoactive ingredient in the brew ayahuasca. In this report, we performed a review of the surrounding literature and detail two deaths which recently occurred in the UK following DMT use. A literature search of both academic (PubMed, GoogleScholar) and media (using Google search engine) publications was performed to identify previously reported fatalities following DMT use. The National Programme on Substance Use Mortality (NPSUM) was also searched for deaths which have occurred in the UK following DMT use. Literature search—There have been three previous reports of fatalities following DMT use, all deemed accidental in nature, with DMT consumption taking place as part of an ayahuasca ceremony in two of these cases. NPSUM cases—Two cases were identified (Case Report 1 [CR1] & Case Report 2 [CR2]), neither of which occurred in the context of an ayahuasca ceremony. DMT was detected and quantified in femoral blood in both cases (CR1 0.23 mg/l; CR2 0.24 mg/l). There was evidence of polydrug use in both cases (CR1 *n* = 6; CR2 *n* = 9), which in each case included additional compounds which can increase serotonergic drive (CR1 cocaine, amphetamine; CR2 venlafaxine, mirtazapine). There have been two recent deaths following DMT use in the UK, both in the context of polydrug use which may have caused death due to excessive serotonergic innervation leading to serotonin syndrome. Polydrug use is increasingly common in the UK, and users of unregulated drugs should caution their use in combination with other unregulated drugs and also any prescribed medications.

## Introduction

*N, N*-Dimethyltryptamine (DMT) is a powerful short-acting hallucinogenic compound found in the South American *­Psychotria viridis* plant, and is the major psychoactive ingredient in the brew ayahuasca, commonly used by indigenous Amazonian tribes [1]. Due to the reported ability of DMT to induce spiritual and intellectual insights, there has been a rise in ‘ayahuasca tourism’ in South America [2] and parallel increased use on the wider recreational drug market [3], including in the UK [4] where it is a Category A controlled substance under the Misuse of Drugs Act (1971). At the same time, there has been an emergence of clinical trials investigating the therapeutic benefits of DMT [2].

DMT is a simple tryptamine of the indoleamine hallucinogen group. These compounds have affinity for serotonergic receptors, inducing psychotropic effects through agonism of serotonin type 2A (5-HT_2A_) receptors [1]. If orally administered on its own, DMT is oxidatively deaminated into an inactive metabolite, 3-indole-acetic acid, by the monoamine oxidase A (MAO-A) enzyme during first pass metabolism, rendering DMT inactive [1]. It is therefore common practice to orally administer DMT alongside β-carboline alkaloids such as harmaline and harmine, both potent MAO inhibitors (MAOIs), which prevent extensive DMT degradation, thus maintaining its psychoactivity. In an ayahuasca beverage these are sourced from the bark of *Banisteriopsis caapi* [4] or infused into smokable plant matter alongside DMT crystal freebase to form *changa* [5].

Subjective effects of the drug include perceptual and cognitive changes, auditory and visual hallucinations/disturbances and mood alterations [1, 6]. Smoking or vaporizing represent the most common methods of recreational administration [6], as this route produces effects almost instantly and which last between 15 and 30 minutes. Oral consumption, typically seen in a more traditional ceremonial setting, takes longer to produce effects (30–60 min). but these last for much longer (3–7 hours). A typical smoked dose of DMT is between 40 and 50 mg (0.6–0.7 mg/kg) [1], whilst an average ceremonial oral dose of ayahuasca is 100–150 mL liquid containing 1 mg/kg DMT, 1.7 mg/kg harmine, and 0.11 mg/kg harmaline [2].

Recent investigations into the adverse events, safety profile and toxicity of ayahuasca report minimal physical and psychological sequelae, with vomiting and nausea found to be the most common adverse events [2]. Clinical studies administering DMT to healthy volunteers report cardiovascular events of elevated blood pressure, heart rate and raised temperature [1]. Studies in rats have shown the lethal dose of DMT to be 50 times the average ceremonial dose, whilst other rodent studies have shown a human equivalent LD50 to be 20 times [2]. Consequently, there are few reported cases of DMT fatalities.

However, the interaction between the serotonergic agonist activity of DMT, the MAOI activity of harmala alkaloids and the serotonin reuptake inhibitor activity of some antidepressant classes (selective serotonin reuptake inhibitors [SSRIs], serotonin and noradrenaline reuptake inhibitors [SNRIs], tricyclic antidepressants [TCAs]) is a potential source of concern [4].The blocking of serotonin reuptake into presynaptic nerve terminals can cause excessive serotonin concentrations to accumulate, causing the potentially fatal serotonin syndrome. Recreational drugs which block serotonin reuptake, such as cocaine, have also been implicated in serotonin syndrome [7], therefore concomitant use with DMT and harmala alkaloids may also increase the possibility of this condition occurring.

Serotonin syndrome symptoms range from mild- nausea, anxiety and insomnia, through to life-threatening neuroexcitation, delirium, and extreme fluctuations in the cardiovascular system [7]. Milder cases of serotonin toxicity can be treated with oxygen and IV fluids, whilst more severe cases require emergency care treatment and pharmacological intervention, with the use of 5-HT_2A_ antagonist cyproheptadine indicated [8].

Here we review the literature and describe two recent fatal cases involving recreational use of DMT and their toxicological findings.

### Case report 1 (CR1)

The deceased was a 36-year-old male with no medical history of note or active prescriptions. On the day of death, the deceased was at a friend’s house where he was reported to have taken DMT. Following ingestion, he collapsed, CPR was attempted, but was pronounced deceased. Compounds which were listed as available to the deceased included herbal material thought to be cannabis, light brown powder in a snap bag and white powder in a snap bag. An inquest concluded that cause of death was due to cocaine and DMT toxicity, with secondary causes of coronary atherosclerosis and left ventricular hypertrophy.

### Case report 2 (CR2)

The deceased was a 58-year-old male with a history of depression and hypertension. Active prescriptions included amlodipine, ramipril, propranolol, mirtazapine, venlafaxine, prochlorperazine, metoclopramide and diazepam. On the day of death, paramedics were called to the deceased’s home address after he was found collapsed and unresponsive in the hallway by his spouse. Paramedics attended but he was pronounced deceased. A search by police at the address found evidence of drug use in the living room including a burnt piece of foil shaped like a spoon. Additionally, a packet of seeds was found, with a possible identification as Syrian Rue (*Peganum Harmala*), although this was unconfirmed. An inquest concluded that the cause of death was due to hypertensive crisis and serotonin syndrome, with secondary causes of drug toxicity and hypertensive heart disease.

## Materials and methods

### Literature search

A literature search was conducted using PubMed and Web of Science with the terms ‘DMT’ or ‘ayahuasca’ and ‘fatal*’ or ‘death’ on 1st November 2024. In addition, searches of the Internet were conducted using the Google search engine using the same terms.

### Case report identification

Case reports were identified by searching the postmortem drug fields of cases received by the National Programme on Substance Use Mortality (NPSUM, previously known as the National Programme on Substance Abuse Deaths [NPSAD]) by 1 November 2024 for the numerical code assigned to DMT. The NPSUM collates voluntary reports from coroners across England, Wales and Northern Ireland pertaining to deaths following psychoactive drug use, as previously described [9].The King’s College London Biomedical & Health Sciences, Dentistry, Medicine and Natural & Mathematical Sciences Research Ethics Sub Committee re-confirmed in August 2024 that research using NPSUM data does not require REC review as all subjects are deceased.

### Toxicological analyses

#### Case report 1

##### General drug screen

Screening of postmortem blood and urine was performed at the Toxicology Unit at the Northern General Hospital in Sheffield in line with the methodology published by Rab et al. [10].

*Sample preparation.* A femoral blood and a urine sample, both preserved with fluoride oxalate, were collected during postmortem examination. They were transported to the laboratory at ambient temperature and then stored at 4 °C until analysis.

*Sample extraction.* 50 µL of postmortem femoral blood was subjected to protein precipitation by dilution (1 + 6, v/v) with ice-cold protein precipitation solution (4 + 1 + 1 methanol, deionized water, 0.1 M zinc sulphate solution). Diluted samples were vortex mixed and centrifuged (3000 rpm, 10 minutes). The supernatant was evaporated to dryness under nitrogen at 40 °C before being reconstituted in 250 µL of a 10% methanol solution in deionized water. Urine samples were diluted (1 + 9, v/v) with 10% methanol solution in deionized water.

*LC conditions:* Liquid chromatography was performed using an UltiMate 3000 high performance liquid chromatography system coupled to a Q-Exactive Focus mass spectrometer (Thermo Scientific, Waltham, MA, USA). The UltiMate 3000 system consisted of a quaternary pump, autosampler and column oven. Chromatographic separation was achieved using a Raptor biphenyl column (2.7 µm, 2.1 × 100 mm, Thames Restek, Saunderton, UK) held at 40 °C. 5 µL of prepared blood or urine was injected onto the column and eluted using gradient separation at 0.4 mL/min. Eluent A consisted of 0.1% (v/v) formic acid and 2 mmol/L ammonium formate in deionized water. Eluent B was 0.1% (v/v) formic acid, 2 mmol/L ammonium formate in methanol: acetonitrile (1 + 1). Starting conditions consisted of 10% eluent B, increasing to 99% B after 7 minutes. This was held for 1 minute before column re-equilibration was achieved following 5 minutes at 10% B.

*MS conditions:* Column eluent was analysed using a Q-Exactive Focus mass spectrometer operating in full scan mode (mass resolution 35 000, mass range 70 to 1000 amu) with confirmation (stepped higher-energy collisional dissociation [HCD] cell settings of 17.5, 30 and 52.3, mass resolution 17 500, mass range 50 to 1000 amu). Ionization was in positive ion electrospray mode with the source operating at a spray voltage of 3500 v, a capillary temperature of 350 °C, auxiliary gas heater temperature of 400 °C, sheath gas flow rate of 35 units, auxiliary gas of 15 and sweep gas of 0. Results were screened using the Tox Explorer^TM^ library (Thermo Scientific, Waltham, MA, USA). Compounds were identified on the basis of the accurate mass of the protonated molecular ion (±5 ppm), a retention time within ±15 s of the target (target defined by prior analysis of a DMT standard), an isotope score % fit threshold of at least 70% and at least one fragment within 10 ppm of its theoretical mass. Two qualifier ions (*m/z* 144.0808, 58.0660) were used for identification of DMT.

##### DMT quantification

Quantification of DMT was carried out at Analytical Services International (ASI; London, UK).

*Sample preparation and extraction.* Liquid chromatography with quadrupole time-of-flight mass spectrometry (LC–QTOF-MS) was used. Seven DMT calibrators (reference material from Merck, Gillingham, UK) were prepared over the range 0.005-1.00 mg/L in analyte-free whole blood. QC samples were independently prepared at two concentrations (0.04 and 0.25 mg/L). Samples (100 µL fluoride-oxalate preserved post mortem blood), calibrators and QCs were prepared by protein precipitation (1 + 4, *v/v*) with acetonitrile and addition of internal standard (amfetamine-d_11_ in methanol, 0.1 mg/L). After vortex mixing and centrifugation, the supernatant was diluted (1 + 1, *v/v*) with Eluent A prior to analysis by LC–QTOF-MS.

*LC conditions.* Analysis of sample extracts was performed utilizing an Agilent Technologies 1290 binary pump, a 1290 Infinity II™ autosampler and column oven (MCT 1290) coupled with a 6545 QTOF MS. Chromatographic separation was performed using an Agilent Poroshell^TM^ EC-C18 column (100 × 2.1 mm, 2.7 µm total particle size) at a temperature of 40 °C, and a constant total flow rate of 0.40 mL/min. Mobile phases consisted of 10 mmol/L ammonium formate in 0.01% (v/v) formic acid in deionized water (mobile phase A) and 10 mmol/L ammonium formate in 0.01% (v/v) formic acid in methanol (mobile phase B). Mobile phase B was also used as the autosampler needle wash solvent. Gradient elution was as follows: 95% A for 1.00 min following injection, followed by a linear ramp to 100% B over 9.00 min. The column was held at 100% B for 2.00 min, prior to re-equilibration at 95% A for 1.00 min. The piston seal wash was 50:50 (v/v) isopropanol: deionized water. Eluent flow was diverted to waste for the first 60 s of each analysis.

*MS/MS and QTOF conditions.* The 6545 QTOF MS was used with a dual-spray electrospray ionization source (Jet Stream™, Agilent Technologies). QTOF-MS data were acquired from 40 to 1000 *m/z* in data-independent acquisition (‘all-ions’) mode. Acquisition rate was 3 scans per second (2693 transients/spectrum) with alternating MS spectra (CE 0 and 30 V, same *m/z* range for both scans). Daily mass calibration was carried out using calibration solution stored on the instrument, and reference ion solution was delivered throughout the analysis via the second nebuliser of the dual spray source at a constant flow rate using a 1260 isocratic pump (Agilent Technologies), flowing at 1.00 mL/min with a 1:100 flow splitter. The total analysis time was 13 min.

*Data processing.* For data processing, chromatograms for the DMT and the IS were extracted from full-scan spectra as protonated ([M + H]^+^) ions using ± 10 ppm extraction windows vs theoretical *m/z* values. Two qualifier ions (*m/z* 144.0805, 58.0652) were included from the 30 V scans for analyte qualification. Instrument control and data acquisition were performed using MassHunter™ (version 10, Agilent Technologies). The assay was partially validated based on local protocols, and the FDA bioanalytical method validation guidelines (US FDA, 2018). Accuracy was 85%–115% of the nominal concentration for calibrators and QCs (80%–120% at the LLOQ, 0.005 mg/L). Calibration curves were fitted with a quadratic curve (1/concentration weighting, *R*^2^ > 0.995). Case samples were analysed in duplicate.

#### Case report 2

All analysis for CR2 was performed in the Toxicology Department at the Royal Sussex County Hospital.

##### General drug screen

*Sample preparation.* Preserved blood and unpreserved urine samples were collected during postmortem examination. They were transported to the laboratory at ambient temperature and then stored at 4 °C until analysis.

*Sample extraction.* Samples were extracted using a method validated in-house for the qualitative detection of a wide range of neutral and basic drugs. 500 µL of preserved (fluoride oxalate) femoral blood or unpreserved urine was added to 2 mL of water, followed by 50 µL of an internal standard mix. Subsequently, 2 g of extraction salt (ammonium sulphate, sodium carbonate, and sodium hydrogen carbonate in a 6:2:1 ratio w/w) was added. An extraction solvent (2.5 mL) consisting of n-heptane, dichloromethane, dichloroethane, and propan-2-ol in a 3:3:3:1 ratio v/v was then introduced. Samples were mixed on a roller mixer for 20 minutes and centrifuged at 1160 g for 5 minutes. The organic layer was transferred into concentrator cups and evaporated to dryness under warm air. Extracts were reconstituted in 150 µL of a 1:1 acetonitrile-water mixture containing 0.1% formic acid. The prepared extracts were transferred to autosampler vials for HR-MS analysis.

*LC conditions.* A Thermo Scientific Vanquish Flex ultra-high performance liquid chromatography (UHPLC) system was used, which included a binary pump, autosampler, and column oven, coupled to a Thermo Scientific Exploris 120 Orbitrap mass spectrometer. Chromatographic separation was achieved using a Thermo Scientific Accucore Phenyl Hexyl column with dimensions of 100 mm × 2.1 mm and a 2.6 µm particle size. The column temperature was maintained at 40 °C throughout the analysis, with a flow rate consistently set at 0.5 mL/min. The mobile phases consisted of an aqueous phase containing 2 mmol ammonium formate in water with 0.1% formic acid, and an organic phase comprising 2 mmol ammonium formate in a 1:1 mixture of acetonitrile and methanol (v/v) also containing 0.1% formic acid. Autosampler needle and seal washes were prepared using a solution of 10% methanol in water.

The chromatographic gradient began with 1% organic phase held for the initial minute of the run. This was ramped up linearly to 99% organic over 10 minutes and held for 1.5 minutes before re-equilibrating back to 1% organic over 4 minutes. The total run time for each injection was 15.5 minutes. To reduce contamination, the first minute and final 3.5 minutes of each run were diverted to waste.

*Mass spectrometry conditions:* Mass spectrometry analysis was conducted on the Thermo Scientific Exploris 120 Orbitrap system, equipped with an OptaMax NG ion source featuring a heated electrospray ionization (ESI) probe. The spray voltage was set at 3500 V for positive mode and −2000 V for negative mode. The ion transfer tube temperature was maintained at 325 °C, while the vaporizer temperature was held at 350 °C. Sheath gas was set to 55 arbitrary units, auxiliary gas to 10 arbitrary units, and sweep gas to 1 arbitrary unit.

In positive ionization mode, analysis began with a precursor scan at a resolution of 60 000 full width at half maximum (FWHM) at  *m/z* 200. This was followed by data-dependent MS/MS acquisition of the top four peaks per scan, using quadrupole isolation at a width of 1.5 mass units. Stepped HCD collision energies of 18.75%, 37.5%, and 56.25% were used to maximize fragmentation coverage. A compound target list was used to prioritize compounds of interest, and if a target was present, MS/MS was carried out. In the absence of a target, the most intense peaks were selected for spectral data collection. Data-dependent MS/MS scans were conducted at a resolution of 15 000. Negative ionization mode followed a similar workflow, starting with a precursor scan and data-dependent MS/MS acquisition of the top two peaks per cycle.

Compounds were identified based on their protonated accurate mass, which had to be within 5 ppm of the theoretical value, and a signal-to-noise ratio exceeding 5. Retention times were required to be within 30 s of the target. Identification also required the presence of at least one key fragment within 10 ppm of its theoretical mass, with isotope pattern scoring used to confirm compound identity.

##### DMT and Harmala alkaloid quantification

*Sample preparation.* Quantification of DMT, Harmine and Harmaline was carried out using a separately developed LC–MS method using standard additions. Reference materials and deuterated internal standard (DMT-d_6_) were obtained from Chiron (Trondheim, Norway). One hundred microliters of preserved blood was spiked with a standard solution containing 100 µg/L of DMT, Harmine and Harmaline with the following volumes: 20, 50, 200, and 500 µl to produce a range of up spikes covering a concentration range of 20 to 500 µg/L. Each sample was also spiked with 50 µl of an DMT-d_6_ at a concentration of 1 mg/L as an internal standard.

*Sample extraction.* Following the preparation of spikes, the samples were then extracted using the same liquid-liquid extraction method as described for the general drug screen.

*LC conditions.* An Agilent 1100 HPLC system including a quaternary pump and autosampler was used for chromatographic separation. Chromatographic separation was performed using a Supelco Discovery HS F5 column (3.3 cm × 2.1 mm, 3 µm particle size) column. Flow rate was set to 600 µl/min. Mobile phase A consisted of Acetonitrile with 0.1% (v/v) formic acid and Mobile phase B consisted of deionized water with 0.1% (v/v) formic acid. Chromatographic conditions were as follows: 15% A for 1.50 minutes following injection with a linear ramp to 90% A over 4.50 minutes. This was held for 2.00 minutes prior to re-equilibration at 15% A for 2.00 minutes.

*MS/MS conditions.* Mass spectral analysis was carried out using an API 4000 QTRAP mass spectrometer. Positive electrospray ionization mode was used. All molecules were analysed by multiple reaction monitoring. MRM transitions and relevant parameters are shown in Table 1. Curtain gas, nebulizing gas (GS1) and Curtain gas (GS2) were set to 30 units each. Electrospray voltage was set to 5500 V and source temperature was set to 500 °C. CAD gas was set to high.

**Table 1.** Multiple reaction monitoring (MRM) transitions for the method

Name	Q1	Q3	DP	CE	CXP
DMT 1	189.1	58.1	56	27	10
DMT 2	189.1	144.1	56	27	10
DMT-d6	195.0	144.1	45	25	10
Harmine 1	213.0	170.0	71	43	30
Harmine 2	213.0	198.0	71	33	12
Harmaline 1	215.0	200.0	71	33	12
Harmaline 2	215.0	172.0	71	43	30

CE = collision energy; CXP = collision cell exit potential; DP = declustering potential.

**Table 2.** Fatal DMT case reports from the literature

Case #	Country and year	Age Sex	Circumstances	Analysis methods	Biological samples and concentrations (mg/L) detected	Cause of death	Other drugs detected and concentrations (mg/l) where quantified	Non-biological exhibits
A	USA 2005 [13]	25 M	Deceased drank ‘herbal tonic’ before going to sleep and was found dead in bed the next morning. No past medical history indicated.	Liquid chromatography Mass spectrometry	Heart blood DMT 0.02 Harmaline 0.07 Harmine 0.17 Peripheral blood DMT 0.01 Harmaline 0.04 Harmine 0.08 Gastric contents DMT 3.3 Harmaline 6.4 Harmine 121.9 Bile DMT 0.57 Harmaline 0.41 Harmine 1.64 Urine DMT 0.89 Harmaline 2.26 Harmine 1.15	Hallucinogenic amine intoxication	Heart blood 5-MeO-DMT 1.88 Urine 5-MeO-DMT 9.59 Diphenhydramine	Eyewitness testimony
B	UK 2014 [12]	M	Attended a shaman led ceremony where he ingested ayahuasca, became agitated and required restraining, later became comatose and died 4 days later. No medical history indicated.	Not specified	No quantitative concentrations available but ‘the ingestion of ayahuasca was inferred by the presence of DMT in the toxicological analysis’ [of the gut]	Inconclusive	Psilocybe spores, cannabis pollen & opium poppy seeds- detected under microscope in ileum and colon	Flasks, containers and drawers found in deceased’s bedroom containing various pollens, plant spores and fungi
C	Colombia 2014 [11]	19 M	British backpacker ingested ‘3 cups’ of yage during tribal ritual, then 2 days later ingested one more, fell ill and breathing slowed, taken to hospital but died on way. No medical history of note.	Not specified	Not provided	Intoxication by ayahuasca and hyoscine	Hyoscine (scopolamine) also ingested (tissues unspecified)	Eyewitness testimony

**Table 3.** Fatal DMT case reports in the present study

Case #	Country and year	Age Sex	Circumstances	Analysis methods	Biological samples and concentrations (mg/L) detected	Cause of death	Other drugs detected and concentrations (mg/l) where quantified	Non-biological exhibits
CR1	UK 2022	36 M	Collapsed at friend’s home after taking DMT, CPR attempted but died at scene. No medical history of note.	Liquid chromatography High resolution mass spectrometry	Femoral Blood DMT 0.23 Urine DMT detected	1a. Cocaine and DMT toxicity 2. Coronary atherosclerosis and left ventricular hypertrophy	Femoral Blood Cocaine 0.29 Benzoylecgonine 0.97 Ethanol 83 Urine Ethanol 130 Cocaine Benzoylecgonine Cannabinoids Amphetamine Paracetamol all detected	Herbal material, light brown powder in a snap bag, white powder in a snap bag
CR2	UK 2023	58 M	Found unresponsive by wife at home, pronounced deceased by attending paramedics. History of depression and hypertension.	Liquid chromatography High resolution mass spectrometry	Femoral Blood DMT 0.24 Harmaline 0.046 Harmine 0.017 Urine All detected	1a. Hypertensive crisis and serotonin syndrome 1b. Drug toxicity 2. Hypertensive heart disease	Femoral Blood Ketamine 0.59 Diazepam Venlafaxine Mirtazapine Propranolol Codeine Paracetamol all detected Urine Diazepam Venlafaxine Mirtazapine Propranolol Codeine Morphine Paracetamol Ketamine all detected	Burnt piece of foil shaped into spoon, packet of seeds (possibly Syrian Rue)

*Data processing.* Peak area ratios of the analyte to the internal standard were calculated for each spiked level, and a calibration curve was constructed. The x-intercept of the calibration curve, taken as the inverse of its negative value, was used to determine the concentration of DMT, harmine and harmaline in the unspiked sample. This method was validated using local protocols based on published guidance (Hasegawa K, Minakata K, Suzuki M, Suzuki O (2021) The standard addition method and its validation in forensic toxicology. Forensic Toxicology 39:311–333) The standard addition calibration curve for each analyte had an r^2^ value of >0.99. Replicate analysis was not carried out due to sample volume limitations but uncertainty within the SAR was estimated, with %RSD of DMT of 2.4% and harmine and harmaline showed higher uncertainty, but still less than 15%. LOD and LOQ were not formally assessed, but the signal of the unspiked sample was significantly greater than that used to define LOQs.

The foil identified at the scene was also swabbed and sent to a separate laboratory for testing.

## Results

The literature review identified 3 prior cases of death involving DMT use (Table 2).

**Figure 1.**
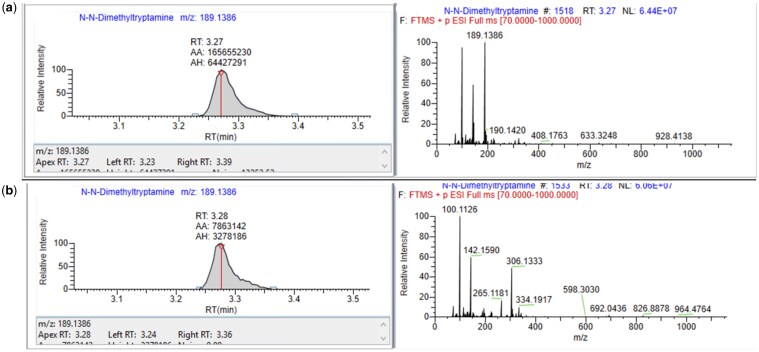
Extracted ion chromatograms showing DMT (C12H16N2, [M + H]^+^ m/z 189.1386 ± 5 ppm, retention time 3.28 minutes) in (a) postmortem blood and (b) urine, and corresponding MS spectra obtained using full scan mode (scan range 70 to 1000 m/z, automatic gain control target 1e^6^).

**Figure 2.**
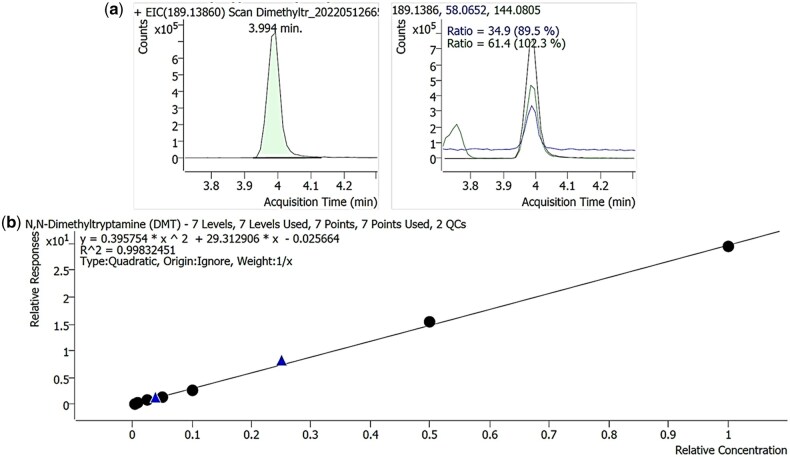
(a) Extracted ion chromatograms for DMT and qualifier ions and (b) DMT calibration curve.

**Figure 3.**
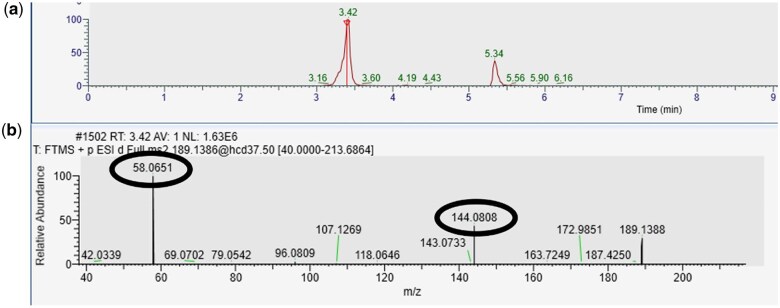
LC–HRAM-MS data for *N*, *N*-dimethyltryptamine from an extracted preserved blood sample. (a) Extracted ion chromatogram for *N*, *N*-dimethyltryptamine (C12H16N2, [M + H]+ m/z 189.1386 ± 5 ppm, retention time 3.42 mins). The peak at 5.34 mins is an unknown. (b) MS/MS spectra for the *N*, *N*-dimethyltryptamine peak measured at m/z 189.1386. Black rings highlight the key fragments m/z 58.0651 and m/z 144.0808.

Traces showing detections of DMT from CR1 can be seen in Fig. 1, with the chromatograms and calibration curve for their quantification in Fig. 2. Traces showing detections of DMT from CR2 can be seen in Fig. 3. CR1 and CR2 are summarised in Table 3.

## Discussion

These are the first reported fatalities in the UK with DMT being confirmed as a cause of death. One previous UK fatality occurred in 2014 [12] after the decedent attended an ayahuasca ceremony, however an inconclusive verdict was returned by the coroner due to the presence of other psychotropic substances detected in the decedent’s gastric contents.

The concentrations of DMT in the postmortem blood for CR1 and CR2 were 0.23 and 0.24 mg/L respectively, which are much higher than that reported in the only other fatal case where a DMT quantification was performed (0.02 mg/L) [13], but comparable to the peak plasma concentration achieved (0.204 mg/L) when volunteers were intravenously dosed with 0.4 mg/kg DMT (similar to a recreational dose) [14]. However, as DMT is eliminated rapidly with clearance rates between 7.5–48.8 L/min and half-life durations of 4.8–19 minutes [15], and the precise time between DMT administration and fatality is unknown in the case reports presented here and the case from the literature where a DMT quantification was performed, it is difficult to ascertain what a fatal DMT dose may be. Furthermore, as DMT is a highly lipophilic molecule (logP 2.573) [1] with *in vivo* human studies showing a majority (67.7%) remains unbound in plasma [16], DMT is likely subject to a high degree of postmortem redistribution further complexifying the determination of a fatal DMT dose.

In CR2, the burnt tin foil tested positive for traces of DMT, signifying this was possibly used to vaporize and inhale DMT. Smoking or vaporizing DMT is the most popular method of administration as the oral bioavailability of DMT is low due to its rapid first pass metabolism [6], but it is difficult to dose or ascertain how much is ingested. Similarly, ayahuasca preparations can vary in DMT and alkaloid content, due to different extraction techniques employed on a variety of plants [1]. The harmala alkaloids quantified detected in CR2 could be attributable to the Syrian Rue seeds found at the scene; these seeds can be prepared into a tea or also smoked to activate their MAOI effects [17]. The concentrations of harmala alkaloids detected were similar to those of peak plasma concentrations found when orally dosing volunteers with a typical ayahuasca brew [18, 19]. The similarities between the concentrations indicated in the toxicology reports and data from controlled studies indicate that both fatalities were unlikely due to significant recreational dosing errors of the DMT itself.

DMT has been detected endogenously in mammalian species, albeit in small quantities, although its functional role remains unclear [20]. Previous investigations into quantifying endogenous DMT concentrations in blood found a range between 0.05 and 55 µg/L [20], therefore the concentrations of DMT present in the case studies presented here cannot be attributed to endogenous DMT alone.

All other drugs detected in case CR2 were not present in excess or at concentrations attributable to death on their own. The SSRIs (venlafaxine and mirtazapine) in CR2 were confirmed as prescribed to the decedent and detected at concentrations indicative of adherence to therapy. The presence of cocaine in PM blood in CR1 indicated that cocaine use occurred shortly before death, however it was detected at concentrations towards the low end of ranges normally found in fatalities. Although not listed specifically as the cause of death in CR1, the possibility of serotonin syndrome contributing to death was discussed within the coroner’s report. Within the literature, two cases co-detected other substances with affinity for serotonin receptors; 5-methoxy-DMT (Case A) and psilocybe (Case B), although there was no discussion on serotonin syndrome within either report. Fatal cases of serotonin syndrome are rare [21] although misdiagnosis is common due to the nonspecific nature of the presenting clinical symptoms [22], therefore underreporting of cases is likely [23]. However, numbers of cases have been rising globally, due to the prescribing prevalence in clinical practice of pro-serotonergic drugs [22, 23]. Poly-drug use contributes to the risk due to the high number of drugs potentially implicated in serotonin syndrome [21], with fatalities more likely to occur via exposure to multiple serotonergic agents [23, 24]. Combinations which include MAOIs are often cited as causing the highest levels of harm on the serotonin toxicity spectrum [8, 22, 25] ,therefore the addition of these agents (including harmala alkaloids) alongside DMT use could pose increased risks.

Deaths involving DMT are very uncommon, as highlighted by the literature search, therefore two UK cases occurring in quick succession is unusual. It may be possible that the positive reporting of psychedelic clinical trials is influencing drug use [26], and giving the impression that these types of compounds are safe and have rapid therapeutic effects [27]. Figures from WEDINOS, a UK voluntary drug testing service, appear to support the rise in recreational DMT use with 81 drug samples sent in with DMT being the intended purchase between 2016 and 2024 [28]. The numbers of test requests rose progressively in this time period, from one in 2016 to a peak of 22 in 2024. Only one sample was found not to contain DMT with 4-AcO-DMT detected instead, indicating supply purity of DMT is well conserved. Although uncommon, the risk of serotonin syndrome is a reality within this context, especially if recreational use of DMT is rising and more people are attempting to access ayahuasca ceremonies. This risk is further heightened upon co-ingestion of prescribed antidepressants or other unregulated drugs which also act to increase serotonergic transmission (e.g. cocaine, MDMA, amphetamine).

However, organizers of traditional Amazonian ayahuasca ceremonies insist that before a participant can ingest the brew, they must conduct a ‘wash-out period’ of abstaining from any substances (prescribed and non-prescribed) with possible or known interactions with the constituents of ayahuasca [29]. Increased plasma tyramine concentrations can interact with the MAOIs causing an increase in cardiovascular activity, therefore dietary restrictions are placed on tyramine-containing foods. Indeed, a history of hypertension was reported in CR2, with the decedent prescribed anti-hypertensive medication, and hypertensive crisis listed on the cause of death. As these rituals have become appropriated and expanded for the increased demand, these essential safety measures may have ceased being enforced, and such strict directives are also unlikely to be followed by the recreational drug using population.

## Limitations

As DMT is not always tested for in postmortem samples and the NPSUM is reported to voluntarily, it is possible that further deaths following DMT consumption have occurred in the UK than those reported here.

## Conclusion

Deaths from DMT are rare. However, there is a clear user base in the UK and the risk of death is a real possibility when DMT is used in combination with other substances which increase serotonergic neurotransmission. No case study with quantified PM toxicological concentrations reported excess concentrations of DMT, but all case studies described involvement of other substances alongside DMT which contributed to death. These findings appear to support the growing body of literature advising caution around such drug combinations [4, 8, 30].The bourgeoning enthusiasm around psychedelic therapies may be fuelling demand for these associated compounds, therefore honest communication around their potential for harm should be strengthened.

## Data Availability

The data underlying this article will be shared upon reasonable request to the corresponding author.

## References

[1] Brito-da-Costa AM , Dias-da-Silva D, Gomes NGM et al Toxicokinetics and toxicodynamics of ayahuasca alkaloids N, N-dimethyltryptamine (DMT), harmine, harmaline and tetrahydroharmine: clinical and forensic impact. Pharmaceuticals 2020;13:334. 33114119 10.3390/ph13110334 PMC7690791

[2] White E , Kennedy T, Ruffell S et al Ayahuasca and dimethyltryptamine adverse events and toxicity analysis: a systematic thematic review. Int J Toxicol 2024;43:327–39. 38363085 10.1177/10915818241230916 PMC11088222

[3] Memphis Seizes nearly 18 Kilos of Psychedelic Drug DMT in Wood Bark [press release]. US Customs and Border Protection 2021.

[4] James CC , Charles SG. Ayahuasca preparations and serotonin reuptake inhibitors: a potential combination for severe adverse interactions. J Psychoactive Drugs 1998;30:367–9. 9924842 10.1080/02791072.1998.10399712

[5] Pascal M , Luke D, Robinson O. Smokable ‘Vine of the Dead’: two case studies of experiencers of both changa and near-death experiences. Int J Transpers Stud 2024;94:1–24

[6] Winstock AR , Kaar S, Borschmann R. Dimethyltryptamine (DMT): prevalence, user characteristics and abuse liability in a large global sample. J Psychopharmacol 2014;28:49–54. 24284475 10.1177/0269881113513852

[7] Volpi-Abadie J , Kaye AM, Kaye AD. Serotonin syndrome. Ochsner J 2013;13:533–40. 24358002 PMC3865832

[8] Malcolm B , Thomas K. Serotonin toxicity of serotonergic psychedelics. Psychopharmacology (Berl). 2022;239:1881–91. 34251464 10.1007/s00213-021-05876-x

[9] Roberts E , Copeland C, Robson D et al Drug-related deaths associated with vaping product use in the United Kingdom. Addiction 2021;116:2908–11. 33751729 10.1111/add.15468

[10] Rab E , Martin S, Freemont A et al Simultaneous screening and quantitation of drugs and their metabolites in postmortem samples by liquid chromatography-high-resolution mass spectrometry: does it provide any benefits? J Anal Toxicol 2023;47:317–23. 36805935 10.1093/jat/bkad011

[11] Morris S. Briton, 19, died after taking hallucinogen in Colombia, inquest told. The Guardian, 2018.

[12] Patricia EJW , David LH, Kevin JE. Light microscopy can reveal the consumption of a mixture of psychotropic plant and fungal material in suspicious death. J Forensic Legal Med 2015;34:73–80. 10.1016/j.jflm.2015.05.010 26165663

[13] Sklerov J , Levine B, Moore KA et al A fatal intoxication following the ingestion of 5-methoxy-N, N-dimethyltryptamine in an ayahuasca preparation. J Anal Toxicol 2005;29:838–41. 16356341 10.1093/jat/29.8.838

[14] Strassman RJ , Qualls CR. Dose-response study of N, N-dimethyltryptamine in humans. I. Neuroendocrine, autonomic, and cardiovascular effects. Arch Gen Psychiatry 1994;51:85–97. 8297216 10.1001/archpsyc.1994.03950020009001

[15] van der Heijden KV , Otto ME, Schoones JW et al Clinical pharmacokinetics of N, N-dimethyltryptamine (DMT): a systematic review and post-hoc analysis. Clin Pharmacokinet 2025;64:215–27. 39838235 10.1007/s40262-024-01450-8 PMC11782443

[16] Good M , Joel Z, Benway T et al Pharmacokinetics of N, N-dimethyltryptamine in humans. Eur J Drug Metab Pharmacokinet 2023;48:311–27. 37086340 10.1007/s13318-023-00822-y PMC10122081

[17] Yuruktumen A , Karaduman S, Bengi F et al Syrian rue tea: a recipe for disaster. Clin Toxicol 2008;46:749–52. 10.1080/15563650701323205 18803088

[18] Callaway JC , Raymon LP, Hearn WL et al Quantitation of N, N-dimethyltryptamine and harmala alkaloids in human plasma after oral dosing with ayahuasca. J Anal Toxicol 1996;20:492–7. 8889686 10.1093/jat/20.6.492

[19] Callaway JC , McKenna DJ, Grob CS et al Pharmacokinetics of Hoasca alkaloids in healthy humans. J Ethnopharmacol 1999;65:243–56. 10404423 10.1016/s0378-8741(98)00168-8

[20] Barker SA , McIlhenny EH, Strassman R. A critical review of reports of endogenous psychedelic N, N-dimethyltryptamines in humans: 1955–2010. Drug Test Anal 2012;4:617–35. 22371425 10.1002/dta.422

[21] Anthony S , Kevin RS, Alex K et al High risk and low prevalence diseases: serotonin syndrome. Am J Emerg Med 2022;61:90–7. 36057215 10.1016/j.ajem.2022.08.030

[22] Boyer EW , Shannon M. The serotonin syndrome. N Engl J Med 2005;352:1112–20. 15784664 10.1056/NEJMra041867

[23] Sanjay P , Chaturbhuj R, Kaushik R et al Fatal serotonin syndrome: a systematic review of 56 cases in the literature. Clin Toxicol 2021;59:89–100. 10.1080/15563650.2020.1839662 33196298

[24] Frank C. Recognition and treatment of serotonin syndrome. Can Fam Physician 2008;54:988–92. 18625822 PMC2464814

[25] Dunkley EJC , Isbister GK, Sibbritt D et al The Hunter Serotonin Toxicity Criteria: simple and accurate diagnostic decision rules for serotonin toxicity. QJM 2003;96:635–42. 12925718 10.1093/qjmed/hcg109

[26] Petranker R , Anderson T, Farb N. Psychedelic research and the need for transparency: polishing Alice’s looking glass. Front Psychol 2020;11:1681. 32754101 10.3389/fpsyg.2020.01681 PMC7367180

[27] Beiner A. I took part in a radical psychedelic clinical trial and it changed my life forever. The Standard, 2023.

[28] WEDINOS. https://www.wedinos.org/sample-results.

[29] Ruffell SGD , Netzband N, Tsang W et al Ceremonial ayahuasca in amazonian retreats-mental health and epigenetic outcomes from a six-month naturalistic study. Front Psychiatry 2021;12:687615. 34177670 10.3389/fpsyt.2021.687615 PMC8221532

[30] Halman A , Kong G, Sarris J et al Drug–drug interactions involving classic psychedelics: a systematic review. J Psychopharmacol 2024;38:3–18. 37982394 10.1177/02698811231211219 PMC10851641

